# Study and Characterization of Special Gypsum-Based Pastes for Their Use as a Replacement Material in Architectural Restoration and Construction

**DOI:** 10.3390/ma15175877

**Published:** 2022-08-25

**Authors:** María Paz Sáez-Pérez, Jorge A. Durán-Suárez, Amparo Verdú-Vázquez, Tomás Gil-López

**Affiliations:** 1 Department of Architectural Constructions, Advanced Technical School of Building Engineering, University of Granada, c/ Severo Ochoa s/n, 18071 Granada, Spain; 2 Department of Sculpture, Faculty of Fine Arts, University of Granada, Andalucía s/n. Edif. Aynadamar, 18071 Granada, Spain; 3 Departamento de Tecnología de la Edificación, Escuela Técnica Superior de Edificación, Universidad Politécnica de Madrid, 28040 Madrid, Spain

**Keywords:** replacement pastes, water glass, pigments, chromatic evaluation, construction, cultural heritage

## Abstract

Within the construction sector, the use of gypsum-based pastes features in the majority of monuments, giving this material significant relevance in conservation and restoration projects affecting the world’s cultural heritage. In this research, we evaluated special gypsum-based colored pastes mixed with air lime, hydraulic lime and sodium silicate, and eight different pigments for their use as replacement materials in architectural restoration and construction. We analyzed the suitability of their physical and chemical properties and their hydric characteristics, mechanics and colorimetric implications in two different studies after 28 days and 120 days. The characterization of the products has mainly confirmed the suitability of the pastes containing pigments for use in the most common applications for these kinds of mixes, highlighting that their specific capacities are worth leveraging. The crystallization of gypsum minerals, observed in all of the mixes, helps to consolidate the shrinkage cracks which appear inside the pastes, improving their mechanical strength values. Another observation of the pastes is related to the amorphous silica precipitates in the mixes which contained sodium silicate: the latter provided to them good mechanical behavior. The improvement observed in the pastes containing the green earth pigment is substantial, due to the inclusion of aluminum silicates and Mg, which is partly responsible for the increased compressive strength of the pastes. Finally, the colorimetric analysis is of vital importance in determining the loss of intensity of the colors of the pastes used, since subjective observation leads to serious errors of interpretation.

## 1. Introduction

Plaster is a commonly-used material due both to its abundance and the fact that it is easy to extract, transform and distribute [1]. In addition, there are other features, such as its easy preparation, durability and versatility [2], along with its quick setting and hardening when exposed to air. It is a material that is often used in the construction sector due to its low cost, its excellent thermal insulation and soundproofing properties, its high flame resistance and its low energy consumption during the production process [3,4]. It is also easy to recycle using suitable preparation processes based on the theory of hydration and dehydration [5].

In building construction, plaster is used for decorative trimmings and coatings of walls and ceilings; for stucco work and rendering; in interiors and exteriors; as a binder in ceramic and stone materials; and even for building products such as bricks and blocks, laminated plasterboards, plasterboard sandwich panels, etc. [6,7].

Despite its aforementioned advantages, its extreme fragility and limited mechanical strength and resistance to water mean that this material is unsuitable when its use requires it to withstand certain specific stresses, to bear shock loads or to be located in external environments [8]. 

For some time, research has been being carried out into how to improve the mechanical properties of pastes so as to broaden the scope of their use. This fact has led to new mixes being studied in which natural and artificial fibers are added [9,10,11,12,13,14,15,16]. Usually, in the case of plasters or gypsum-based pastes, the way that this is achieved is by reinforcing them using fiberglass [17,18,19].

The search for materials which have lower environmental impacts and greater efficiency has led to artificial fiber-based additives being replaced by others containing natural fibers [3]. However, the high cost of natural fiber in comparison with that of plaster, along with the limited interaction of these additives with the plaster matrix, mean that these compounds are not as competitive as artificial ones.

Within the construction sector, the use of gypsum-based pastes is necessary for the majority of monuments, giving this material significant relevance in conservation and restoration projects affecting the world’s cultural heritage. Its origin dates back to the sixth millennium BC in Greece, and it was widely used and developed by the Romans [20]. Consequently, knowledge of this type of compound, and its application, is fundamental for mitigating the state of degradation and loss of built heritage [21,22,23,24]. Despite its importance, there has been little interest in studying it or defining new strategies for its use in conservation for a number of years.

Fortunately, in recent decades, the features of mortars and pastes have become a priority in material characterization studies, particularly those which have compositional or microstructural issues [25,26,27,28,29,30,31,32,33,34], excluding aesthetic matters [21,35,36,37,38]. In this regard, incorporating polymers in traditional building materials, such as mortars, adds great value when compared to conventional building materials. The addition of polymers makes it possible to obtain good levels of mechanical strength, good adhesion properties, abrasion and weathering resistance, waterproofing and excellent insulating properties [39].

New research projects seek to increase the durability of plaster pastes exposed to atmospheric influences. Recent studies have analyzed the improvements brought about by introducing modifying additives to the plaster binder, such as: polymeric compositions, fine minerals and nano-disperse components. Zhukov et al. have analyzed the addition of hardening resins to plaster through polycondensation and the application of nano-aggregates [40]. The addition of a polymer to the plaster mix produces a framework of dehydrated crystalline aggregates during the hydration of the plaster, whilst the resin, when it hardens, forms a continuous polymeric matrix. This causes the polymeric plaster to increase its strength over time due to the continuous polymerization of the resin.

Other research projects seek to analyze the influence of micro-aggregates (micro-spheres, hydroxyethyl methyl cellulose polymer and/or aerogel) on the thermal conductivity coefficient and thermal diffusivity. Using these additives makes it possible to reduce thermal conductivity by up to 23%, compared to the unmodified plaster samples [41]. The authors conclude that the polymer provoked a change in the structure of the plaster compound, giving it a lower density and greater porosity.

On the other hand, the porosity increasing can also have some disadvantages, since a key element to take into account in the conservation of gypsum-based pastes is the permeability of the compound [42]. As such, it is necessary to study the porosity, sphericity and pore size distribution, assessing the changes depending on the mortar composition [43]. Recent studies have made progress by using mercury intrusion porosimetry (MIP) and micro-computerized tomography (µCT). Thanks to these techniques, it is possible to visualize pores, air voids, aggregates and binder distributions within a sample [25].

However, when it comes to heritage, the maintenance and preservation of buildings require broader interventions also addressing issues of aesthetic nature. In this respect, the colorimetry rules and studies can be key in the field of conservation and restoration [44,45,46]. Knowledge of the chromatic possibilities of pigments and their techniques is fundamental when the objective is to carry out an intervention for recovery of built heritage [47,48].

In this kind of intervention, where it is not possible to modify the aesthetic characteristics of the element, colorimetry has become a highly useful tool. In an intervention of this type, a correct choice of materials must be made after evaluating the effectiveness of the procedures to be used and the chromatic modifications that can happen. In this way, it is necessary to develop a colorimetric study at least, before and during the preparation of the restorative pastes.

There are recent studies which have already applied the study of colorimetry in the field of monumental heritage, analyzing the performances of treated pigments [49,50,51,52,53,54,55,56]. For this reason, the use of pigments in different applications within the field of materials engineering and interventions in architectural heritage is necessary in order to achieve matching visual and aesthetic characteristics [57,58].

Generally, the colorimetry studies have focused on the characteristics of the pigments, mainly on production processes and their formulation, the saving of resources, the product finish and the most suitable application methods for protecting the environment. However, it is important to underline the conditions that the materials will be expose for establishing their performance and effectiveness.

The present article focuses upon the application of different pigments in plaster-based pastes for use in construction, be it to new builds or to the restoration of cultural heritage sites. It analyzes the intended use of the pigments studied, the suitability of their physical and chemical properties and the characteristics of the materials. Their colorimetric implications were analyzed in two different studies after 28 days and 120 days.

## 2. Materials and Methods

The research carried out involved designing four gypsum-based pastes containing added pigments. Different binders and pigments were used to produce them, making it possible to add significant color to the pastes, and increase their final mechanical strengths without unduly reducing their insulation or water vapor permeability values.

**Figure 1:**
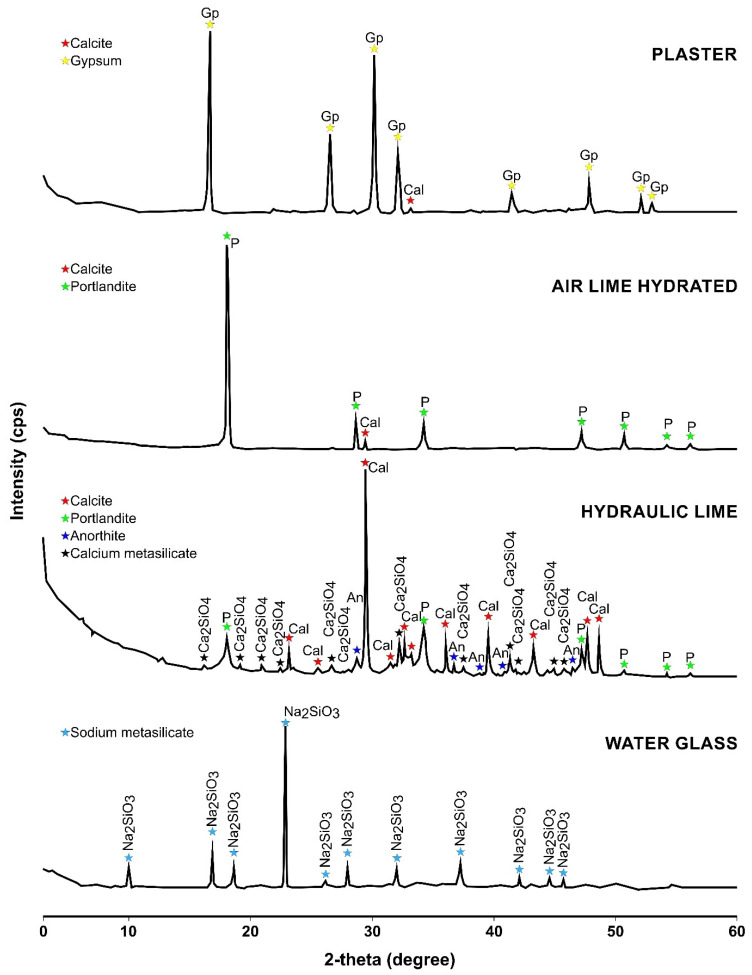
X-ray diffractograms for the plaster, hydrated air lime, hydraulic lime and water glass [60]. Abbreviations for names of rock-forming minerals.

Both the limes and plaster used were provided by CTS Spain. The plaster used contained a minimum of 90% of hemihydrate, giving it the highest quality, as can be seen in the XRD (Figure 1). According to the distributor, a microfiltration process is applied to the air lime after it has been slaked in tanks used specifically for that purpose, and it is then aged for a period of no less than six months. According to [59], air lime is a type CL90 calcium lime (CL), the CaO + MgO content of which is ≥90%. The MgO content is ≤5%, the CO_2_ content is ≤4%, the SO_3_ content is ≤2% and the usable lime (Ca(OH)_2_) content is ≥80%. Where hydraulic lime (NHL5) is concerned, this was genuinely natural pure lime obtained from the calcination of loamy limestone, without additives, at production temperatures of 1200 °C, which according to [59], has a usable lime (Ca(OH)_2_) content of ≥15%, a SO_3_ content of ≤2% and compressive strengths ranging from 5 to 15 MPa. The sodium metasilicate (water glass) was acquired from Alquera Ciencia SL (Spain), with a SiO_2_ content of 26.40 ± 1.50%, Na_2_O content of 8.00 ± 0.60% and water content of 65.60 ± 2.00%. The true density is 38 ± 1.00 Be, and the pH is 12.50 ± 1.00. The mixture has a viscosity of 80 MPa·s.

In relation to the different pigments used (eight), these were supplied by Kremer Pigmente. The color range selected enables the use of a broad spectrum of red, blue, green, ochre and yellow colors, specifically for use in the preparation of pastes, putties and mortars for applications in architectural heritage work. The compositional parameters, by manufacturer, color code [61] and name, are set out in Table 1 and Table 2.

### 2.1. Binder Characterization

In order to study the mineralogical, chemical and colorimetric properties of the binders used in the present research, X-ray fluorescence (XRF), X-ray diffraction (XRD) and standard colorimetric observation methods were used, in line with the CIELab 1976 system.

A compact, high-performance wavelength dispersive X-ray fluorescence spectrometer, the Zetium model (Malvern Panalytical Company, Worcestershire, UK) by the brand PANalytical, was used to perform the X-ray fluorescence test (XRF) at the Scientific Instrumentation Centre of the University of Granada (CIC).

A Bruker D8 DISCOVER diffractometer (Dectris, Baden-Daettwil, Switzerland) with a DECTRIS PILATUS3R 100K-A detector (Dectris, Baden-Daettwil, Switzerland), from the Scientific Instrumentation Centre of the University of Granada (CIC), was used for the XRD test. The Xpowder [64] program (v. 8, Daniel, Granada, Spain) was used to determine the composition.

Figure 1 and Table 3 show the results obtained from the XRD and XRF tests.

The preparation of the samples involved grinding the raw materials in an agate pestle and paste and subsequently sieving them (mesh sieve ASTM N° 45, diameter <0.354mm).

A Konica Minolta CM-2500c Spectrophotometer (I.T.A. Aquateknica, S.A. Valencia, Spain) from the University of Granada was used in order to be able to carry out the colorimetric characterization and calculate the CIELab-1976 chromaticity coordinates [65] of the binders, pure pigments and their mixes after 28 and 120 days. The measurements obtained for diffuse spectral reflectance were in the visible range of 360–740 nm, at 5 nm intervals, with a D65 illuminant at 10°. The specular reflection component was excluded from all of the measurements, following CIE recommendations [66].

### 2.2. Sample Design and Preparation

The research was carried out using four different pastes: the first composed entirely of pure plaster; the second made of plaster and air lime; the third made of plaster and hydraulic lime; and the fourth made of plaster and sodium silicate. In all four cases, the pastes were mixed with the pigments described above.

For the compression and flexural tests, several samples with dimensions of 160 × 40 × 40 mm were prepared in a plastic mold. For the permeability test, the samples dimensions were 40 × 40 × 20 mm, being prepared in a plastic mold.

A dosage was specified in terms of the volumes of the mixture components, the final compositions being those shown in Table 4. In order to prepare the samples, the different components (plaster, lime/plaster + pigment) were dry-mixed, before finally adding potable water in order to facilitate the mixing. For the samples containing plaster and water glass, the plaster binder and pigment were dry-mixed before adding the sodium silicate (liquid). This binder (sodium metasilicate) was purchased commercially in proportions of 25% active material, sodium silicate (Na₂SiO₃), and 75% water. The compaction times in the demountable molds and subsequent demolding were 24 h for all samples. 

All of the samples were placed in a Weiss Technik climatic chamber, ClimeEvent model (Xian LIB Environmental Simulation Industry, Shaanxi Province, China), with the following characteristics: temperature range −42 °C to 180 °C and relative humidity of 10–98%.

The environmental setting and hardening conditions for the PPS, PALS and PHLS mixtures were 22 °C and RH 70% inside of a climatic chamber for 120 days. The samples of the PWGS paste had particular conditions of 60 °C for 24 h in an oven, causing the concretion of the binder, and were subsequently kept in the same environmental conditions (in a climatic chamber) as the rest of the samples.

The average time used for sample setting was 28 days, except in the case of the PWGS mixes, which set after 24 h. All samples were considered to have fully hardened after 120 days.

**Figure 2:**
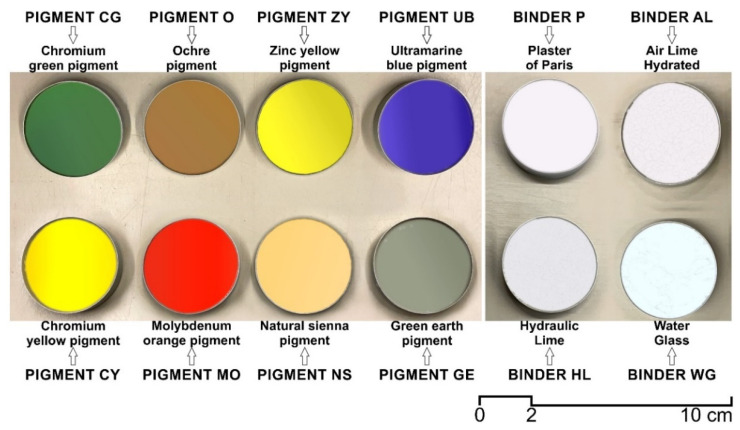
Samples of pigments and binders used in the research.

Figure 2 shows both the pigments and the binders used in the research.

### 2.3. Methods

#### 2.3.1. Scanning Electron Microscopy (SEM)

Using a GEMINI (FESEM) CARL ZEISS scanning electron microscope (SEM) (LEICA, Madrid, Spain), with a Röntec M Series EDX Detector (LEICA, Madrid, Spain), belonging to the Scientific Instrumentation Centre of the University of Granada (CIC) the mineralogical, microstructural and textural characterization of the mixes was performed. The results of the EDX analysis were collected by matrix spotting of the indicated samples.

#### 2.3.2. Water Vapor Permeability

According to [67], sixty-four test pieces were analyzed for each paste type (two for each pigment) and two for each mix without added pigments (eight test pieces), after 120 days, in laboratory conditions (temperature: 20 ± 2 °C; RH: 65 ± 5%). Their dimensions were 40 × 40 × 20 mm. The edges of the samples were sealed using liquid paraffin; then they were placed in plastic recipients with covers, such that one part of the test piece was inside the recipient and the other was outside of it. The join between the test piece and the plastic recipient was sealed using liquid paraffin.

Granular calcium chloride was placed inside the plastic recipients as a drying agent, in sufficient volume in order to obtain a relative humidity of 0%. An air gap measuring approximately 10 mm was left between the drying agent and the base of each test piece.

After preparing the samples, the recipients were weighed in order to determine their initial mass. 

The specimens were brought out from the climatic chamber to measure the weights. The same specimens were used to perform the water vapor permeability tests at both 28 and 120 days.

The test conditions were 50 ± 1% RH and 25 ± 0.5 °C, the samples being weighed every twenty-four hours until the weight difference every twenty-four hours did not exceed 5%.

#### 2.3.3. Mechanical Tests

The flexural strength and compressive strength tests were carried out on each type of test piece, following a hardening time of 120 days. The total of samples used were 216. In order to calculate the strength, regulation [68] was used, for prismatic test pieces with dimensions of 160 × 40 × 40 mm.

The press machine used to perform the break test was IBERTESTEUR TEST MD2 universal testing apparatus (Ibertest, Madrid, Spain). For a sampling interval of 64 mm, the test speed was 1 mm/min. The sample was broken by using a concentrated load on the central part, with the load cell set to 5 kN. In the compression testing, the speed used was 5 mm/min.

Using the average of the results for the three test pieces for each dosage and pigment used, the average mechanical strength value was obtained.

#### 2.3.4. Color Tests

After preparing the samples, their diffuse spectral reflectance curves were measured. Five colorimetric determinations were performed for each one. Using Bessel’s correction, the standard deviation was obtained for the values acquired, without this ever exceeding 3% of the associated average value [69].

Finally, SpectraMagic NX Color Data Software (I.T.A. Aquateknica, S.A., Valencia, Spain) was used to present the simulations of the color variations for the samples studied.

## 3. Results

### 3.1. Scanning Electron Microscopy (SEM)

**Figure 3:**
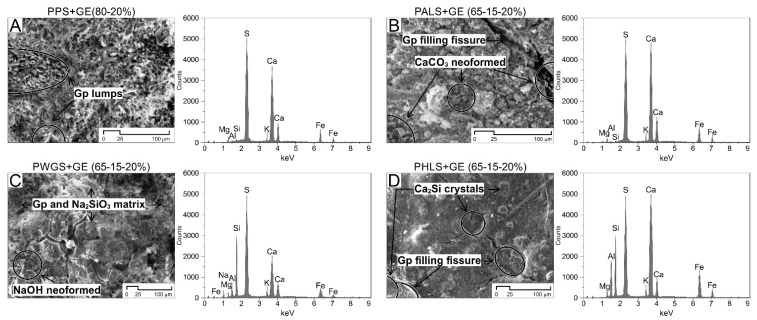
Scanning electron microscopy images corresponding to different pastes with the pigment, GE (Green Earth). The presence of gypsum lumps (**A**), several recrystallizations that are filling cracks (**B**,**D**) and other characteristic mineralization of the paste’s matrix (**B**–**D**) are highlighted. The plot of the EDX analysis corresponds to the elemental composition of the matrix.

Figure 3 shows both the morphological analysis and EDX analysis of the samples.

In the SEM study carried out 120 days after they were prepared, plaster crystallization was detected in specific areas of all of the samples, specifically in cavities with sizes of ≈20–35 µm, along with needle morphology in the case of the PPS + GE sample, where lumps of such crystallization were detected, and in PALS + GE and PHLS + GE samples. It may be speculated that this type of neoformed crystal generates a certain mechanical improvement in the pastes due to the filling and consolidation produced in cracks and micro-fractures.

Meanwhile, calcium carbonate crystallization was found in cavities in some samples, such as those of PALS + GE; belite crystallization (Ca2Si, calcium silicate hydrate) was found in the PHLS + GE samples; and neoformed sodium hydroxide (NaOH) in the plaster and sodium metasilicate matrix of the PWGS + GE samples.

The EDX analyses of the matrixes of all of the samples provided results which are consistent with the quality of the samples. Elements associated with the Green Earth (GE) pigment stood out in all of them. This artificial pigment is obtained by combining two types of hydrous phyllosilicate: celadonite (KMgFe_3_ + Si_4_O_10_(OH)_2_) and glauconite (Fe_3_ +, Al, Mg_2_Si, Al_4_O_10_(OH)_2_). Apart from this, the elements associated with the matrix are those which are characteristic of each mix. S-Ca associations stand out, as they are typical in plaster and air lime (PPS and PALS), along with increases in Si, which is characteristic in samples containing hydraulic lime and water glass.

### 3.2. Water Vapor Permeability Test

**Figure 4:**
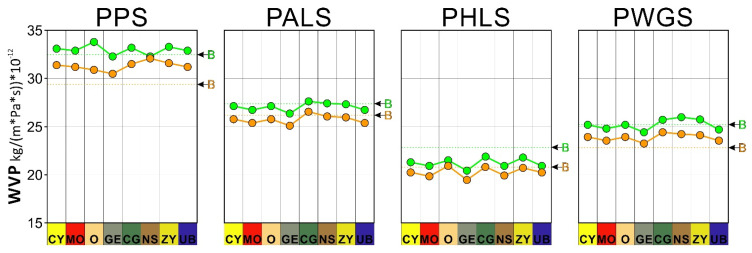
Water vapor permeability of the binders and their corresponding pigmented pastes (PPS, PALS, PHLS and PWGS). The green circles and the orange circles correspond to the permeability at 28 and 120 days respectively. The dotted line and the green letter B correspond to the specific binder at 28 days. The dotted line and the orange letter B correspond to the specific binder at 120 days.

The results of the water vapor permeability tests for both test periods, after 28 and 120 days, are shown in Table 5 and Figure 4.

After 28 days, the average permeability for the PPS + PIGMENT samples was 33kg/(m·Pa·s))·10^−2^. The maximum value was 33.3 ± 1.12kg/(m·Pa·s))·10^−12^ for the sample containing the ZY pigment, and the minimum value was 32.3 ± 1.3kg/(m·Pa·s))·10^−12^ for the sample containing the GE pigment.

In the case of the PALS + pigment samples, the average representative value for the group is 26.83kg/(m·Pa·s))·10^−12^. The maximum value was 27.4 ± 1.33kg/(m·Pa·s))·10^−12^ for the sample containing the CG pigment, and the minimum value was 26.1 ± 1.20kg/(m·Pa·s))·10^−12^ for the sample containing the GE pigment.

The average value of the PWGS + PIGMENT sample group was 24.8kg/(m·Pa·s)) ·10^−12^. There was a maximum value of 25.72 ± 1.8kg/(m·Pa·s)) ·10^−12^ for the NS samples and a minimum value of 24.1 ± 1.11kg/(m·Pa·s)) ·10^−12^ for the GE samples.

Finally, the lowest values were noticed in the plaster and hydraulic lime pastes (PHLS + PIGMENT), the average value being 20.8kg/(m·Pa·s)) ·10^−12^. The maximum value was 21.5 ± 0.94kg/(m·Pa·s)) ·10^−12^ for the CG samples, and the minimum value was 20.0 ± 1.50kg/(m·Pa·s)) ·10^−12^ for the GE samples.

It is deduced from the overall analysis that the substitution of the plaster (−15%) in the different pastes by air lime, sodium silicate and hydraulic lime resulted in lower permeability of the compound. In the different mixes, over time (120 days), it was observed that the values decreased in all cases, confirming compaction of the samples. We recorded reductions in the values of between 8.50% for the PPS + O samples and 0.60% for the PPS + NS samples, for the 28 and 120 days. For the remaining pastes, the values varied between 4% and 5% for each period of study.

### 3.3. Mechanical Tests

**Figure 5:**
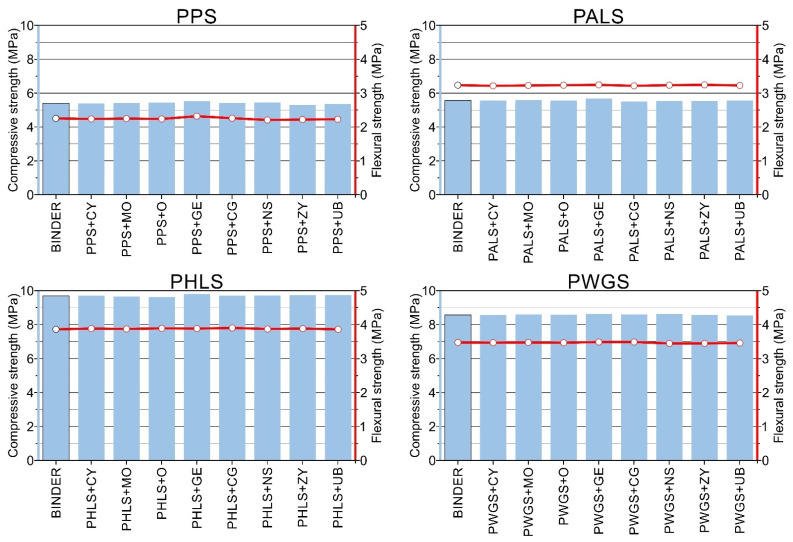
Mechanical strength values (MPa) of binders at 120 days and their corresponding pigment-colored pastes (PPS, PALS, PHLS and PWGS). Blue bar plot corresponds to compressive strength. Line plot with red circles corresponds to flexural strength.

The results obtained in the mechanical tests display differences for the different mixes, in terms of both compressive and flexural strength, during the test period (120 days). The results obtained from the mechanical tests are set out in Table 6 (samples without added pigments) and Table 7 and Figure 5 (binders and samples after 120 days).

#### 3.3.1. Compressive Strength

##### PPS + Pigment Samples

In the plaster paste samples containing added pigment (PPS + PIGMENT), the maximum compressive strength after 120 days was 5.52 MPa ± 0.02 for the PPS + PGE (Green Earth pigment) sample. The minimum value was 5.3 MPa ± 0.05 for the PPS + PZY (Zinc yellow pigment) pigment sample.

In percentage terms, the variation among the pastes containing added pigment was 3.98%, and the difference among the pastes without added pigment was 2.17%. This shows full compatibility between the pastes and the pigments used.

##### PALS + Pigment Samples

The compressive strength of the plaster + lime pastes exhibits a slight increase in the results, compared to the previous group. In this case, the values range between 5.74 MPa ± 0.05 for the PALS + PGE (Green Earth pigment) samples and 5.56 MPa ± 0.03 for the PALS + PCG (chromium green pigment) samples. This represents an average increase of 3.74% compared to the pure plaster samples.

##### PWGS + Pigment Samples

The compressive strength levels of the plaster + sodium silicate pastes were higher compared to the previous groups. In this case, the values range between 8.72 MPa ± 0.01 for the PWGS + PGE (Green Earth pigment) samples and 8.62 MPa ± 0.01 for the PWGS + PUB (Ultramarine Blue pigment) samples. This represents average increases of 37.6% compared to the pure plaster samples and 35.2% compared to the plaster and air lime samples.

##### PHLS + Pigment Samples

The results for this group of samples are the highest of all those tested, reaching maximum values of 9.91 MPa ± 0.02 for the samples containing added pigment (PHLS + PGE (Green Earth pigment) and 9.79 MPa ± 0.03 for the samples without added pigment; percentage differences are less than 1% between them. In relation to the other groups, a significant increase was also confirmed, these being 45% compared to the pure plaster samples (PPS), 42.7% compared to the plaster and air lime (PALS) samples and 11.6% compared to the pastes containing geopolymers (PWGS).

Overall, it was observed that the compressive strength results for the samples containing pigments experienced improvements in resistance capacity, the least resistant being the PPS pastes, followed by the PALS and the PWGS, and the most resistant being the PHLS pastes.

The literature consulted [70,71] confirms that, in general, an increase in compressive strength for this type of paste is the consequence of a variation in the microstructure of the hardened matrix.

Furthermore, [72,73] established that an increase may also be the result of chemical reactions between the components (plaster + hydraulic lime) in the presence of water. The presence of plaster as a replacement for the lime results in faster hydration, and consequently, accelerated setting.

Meanwhile, it was observed that the addition of water glass to the mixes studied resulted in the formation of amorphous silica, which acted as a siliceous aggregate in the resulting paste, thereby increasing the mechanical strength.

Finally, in the samples containing hydraulic lime, an increase in the hydration rate was confirmed due to the presence of calcium silicates, whose role is that of an active additive (catalyst) in these pastes.

The analysis of the samples containing pigments for the four pastes concluded that in all cases, the maximum value was associated with the samples containing the PGE (Green Earth pigment). Previous studies [73,74,75] confirmed that MgO in both the pigment itself and in the binding material acts as a fraction of aggregate material, increasing the compressive strength of the mixes containing added pigments.

#### 3.3.2. Flexural Strength

Where flexural strength is concerned, the results show a very similar tendency to those observed in the compressive strength testing (Table 6 and Table 7). In this case, it is observed that strength increased when part of the plaster was replaced by other binders, such as air lime, sodium silicate or hydraulic lime.

During the period of study, the maximum values were obtained for the plaster + hydraulic lime samples (PHLS samples). Those maximum values were up to 3.94 MPa ± 0.03 (average value of 3.92 MPa). Next were the plaster + water glass samples (PWGS samples), which reached an average value of 3.50 MPa, representing a percentage decrease of 10.7%. In third place were the plaster + air lime samples (PALS samples), which obtained average flexural strength of 2.93 MPa. Finally, the minimum values were achieved by the pure plaster samples (PPS samples), their average value being 2.25 MPa.

In percentage terms, the differences between the groups are significant. The plaster + hydraulic lime samples were 10.7% stronger than the plaster + water glass samples, 25.2% stronger than the plaster + air lime samples and 42.6% stronger than the pure plaster samples.

After comparing the results of the samples containing added pigments, no real difference was observed among them. On comparing the samples without added pigments with the colored samples in each group, it was observed that in the plaster + hydraulic lime samples (PHLS samples) the flexural strength results were very similar to those for the sample without added pigment, there being a difference of <1% in all cases. The samples containing plaster + water glass (GWS sample) obtained similar differences, slightly greater than for the previous group, although still less than <1%. The samples containing plaster + air lime (PALS samples) displayed differences of 2%. Lastly, for the pure plaster samples (PPS samples) the differences were also minor, slightly above 2% (2.17%).

### 3.4. Color Tests

**Figure 6:**
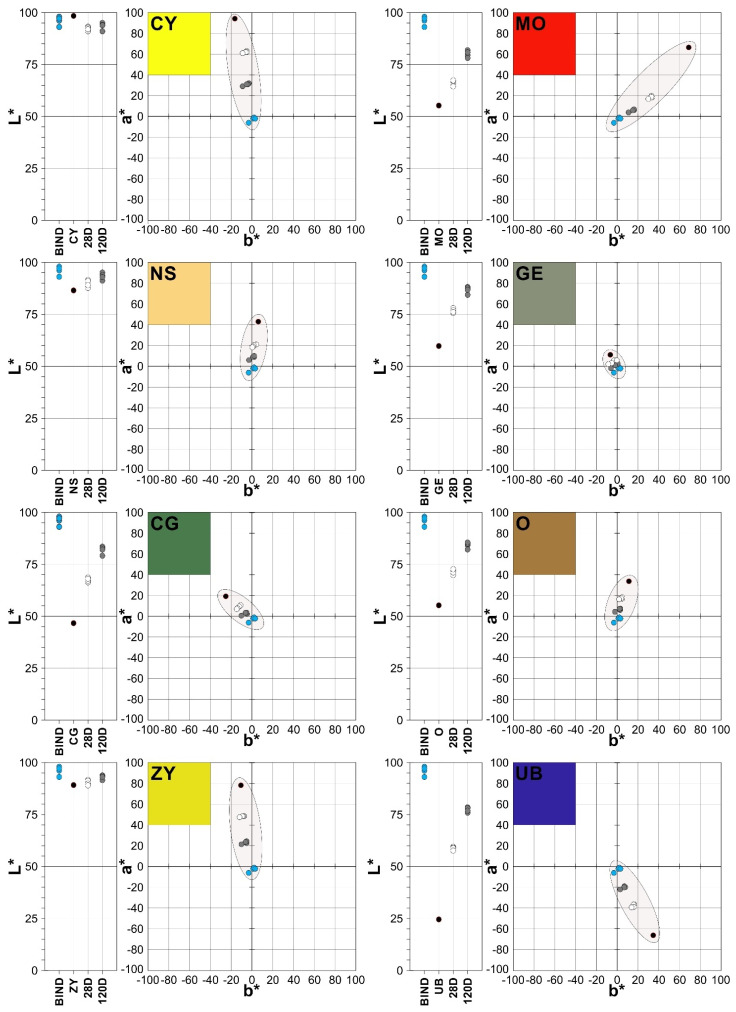
CIELab1976 colour space representation of binders and their corresponding pigment-coloured pastes (PPS, PALS, PHLS and PWGS). Plot L*, indicates brightness values of the different samples. Plot a*-b*, indicates chromaticity values of the different samples. Blue circles are the binder samples without pigment; black circles are the pigment samples; white circles are the paste samples at 28 days; grey circles are the paste samples at 120 days.

Figure 6 shows the color coordinates L*, a* and b* for the mixes studied in the different media tested, in the test phases after 28 and 120 days. Each pigment displays the luminosity values L* on the left and the chromaticity diagram a*-b* on the right. In all cases, the black circles represent the coordinates of the pure pigment, the blue circles the binders, the white circles the pastes after 28 days and the grey circles the pastes after 120 days.

With regard to the binders, they all feature very small tonal amounts (a*-b*) of red and blue, except the water glass, whose tones are blue and green. The luminosities (L*) are extremely high in all cases, with values which are very close to 100%, generating a visual impression that is very similar to white. Meanwhile, the pigments used in all of the mixes in proportions of 20% feature shades which are characteristic of the color palette designed, and they are obviously affected by their interactions with the different binders on forming the pastes.

In terms of shade, the CY pigment is composed of a significant proportion of yellow and also features a considerable amount of green (a*-b*). The luminosity of this chromium pigment is very close to 100%. The luminosity of the pastes hardened for 28 or 120 days tended to differ, darkening in some way by approximately 5%; the color saturation decreased more markedly—by up to 40%.

The MO pigment is largely composed of yellow and red tones (a*-b*), and the luminosity is low: slightly higher than 50%. The changes induced by the different binders increased the luminosity in all cases, this being greater after 120 days, with values of close to 13%. In the chromaticity diagram the saturation of the pastes drastically reduces to levels which are very close to those of the binders (≈80%). Equally noteworthy is the reduction induced by the binders in relation to the yellow shades, at the expense of the associated red shade.

The NS pigment has high luminosity (L*) values, of close to 90%, and where chromaticity (a*-b*) is concerned, yellow stands out as the principal shade alongside small amounts of red. The pastes obtained provoke subsaturation, with values which are close to those of the binders, although the relative reduction was ≈10%. The luminosity increased ≈ 14%. It is notable how there were significant reductions in the yellow and red shades, and the red was replaced by green in the PWGS paste, after 120 days.

The GE pigment has average luminosity (≈60%) and chromaticity values (a*-b*), along with comparatively small amounts of yellow and green. The pastes featured increased luminosity (≈12%), particularly those measured after 120 days, contrary to what happens in the chromaticity diagram, where the pastes display relative subsaturation of ≈8% after 28 and 120 days.

The CG pigment has low luminosity (<50%) and chromaticity components (a*-b*) based on green and yellow shades. The variations in luminosity were significant, particularly the increase in white, which occurred in the pastes after 28 and 120 days (≈15%). The most significant variations in terms of chromaticity stemmed from subsaturation, which arose in the mixes after 28 and 120 days; these have a clear tendency towards the binder values (≈3%).

The O pigment reached luminosity values slightly higher than 50% and tonal proportions (a*-b*) of yellow and red. The changes were similar to those experienced by the rest of the pigments and their pastes: a clear increase in luminosity (≈6%) and a substantial loss of saturation (≈14%). The PWGS sample after 120 days provided a slight green tone compared to the reddish tones of the other samples (PPS, PALS and PHLS).

The ZY pigment has very high luminosity values (≈90%), whilst the chromaticity includes tonal components based on yellow and green. The variations experienced by the pastes on hardening with the binders were very similar to those described in all cases: increased luminosity (≈3%) and loss of saturation (≈30%).

The UB pigment has low L* values, making it dark (≈25%). The changes induced by the inclusion of the binders studied increased luminosity by up to (≈11%) after 120 days. The chromaticity values include a blue tonal component, which dominates in relation to the red tone, which represents half the amount of the blue. The pastes provoked a loss of saturation which left it close to the binders’ values (≈33%).

Table 8 shows that all of the paste samples studied experienced total color variations (ΔE) clearly detectable by the human eye ΔE ≥ 3 [76] after 28 or 120 days. These changes are determined by the chroma variation (ΔC), which entails variations in saturation (subsaturation). This doubled after 120 days in the majority of cases, compared to the amount after 28 days. Likewise, the increased luminosity (ΔL) induced by the binders used to prepare the pastes is a decisive factor in the total color variation (ΔE). The only exception of note occurred in the mixes containing pigment O, whose variations just exceeded the limit of detection by the human eye.

**Figure 7:**
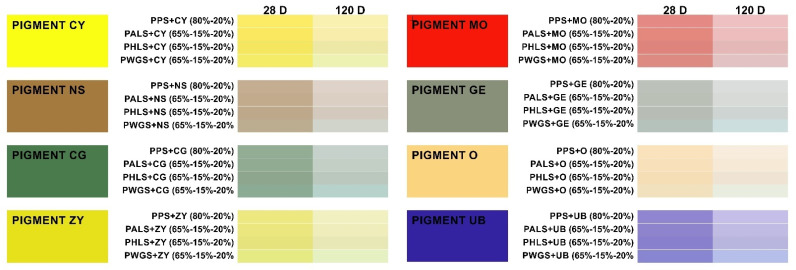
Color chart for the different mixes and inorganic pigments used, with an indication of their color code for both of the test phases (28 days and 120 days).

All of these changes are represented in the form of a color chart in Figure 7, taking into account the average values for a*, b* and L* for each group of samples. For each pigment and paste type, the results are shown for 28 days and 120 days according to their color coordinates (CIELab 1976).

## 4. Conclusions

1. The characterization of the products primarily confirmed the suitability of the pastes containing pigments for use in the most common applications for mixes of this kind.
2. The results obtained in relation to mechanical strength confirmed the increases when air lime and hydraulic lime are used. Likewise, the inclusion of water glass induced improvements in all cases with regard to the samples containing plaster, whilst not outperforming the pastes containing hydraulic lime.
3. From previous studies and the mechanical results obtained, it can be concluded that the presence of magnesium silica aluminates could have been partly responsible for the increases in compressive strength of the mixes containing Green Earth pigment.
4. The crystallization of gypsum minerals, observed in all of the mixes, helped to consolidate the shrinkage cracks which appeared in them, improving their mechanical strength values, just as in the cases of neoformed calcium carbonate and belite originating in PALS and PHLS or sodium metasilicate in the PWGS samples.
5. The water vapor permeability values were high in all cases due to the dominant presence of plaster; nevertheless, the mixes of binders and pigments had reduced values: by around 15% for the pastes after 28 days, and by 25% for the mixes after 120 days.
6. Where the values obtained in the mechanical tests are concerned, the results either coincide with those observed in similar studies [77,78] or are even higher, as in the case of [79]. A similar observation can be made for amorphous silica precipitates in the mixes containing water glass.
7. The colorimetric analysis offerred total color differences in the pastes containing added pigments, compared to the pure pigments, which were clearly detectable by the human eye in all cases, after 28 and 120 days. Pigment O and its mixes were at the limits of visual perception, the ΔE values being close to three. The main modifications were determined by the variations in chromaticity (ΔC), resulting in subsaturation and duplicating that perception in the majority of cases after 120 days. Meanwhile, the variation in luminosity induced by the whitish appearance of the binders was a decisive factor in the perception of the total color of the pastes.

## Figures and Tables

**Table 1:** List of pigments along with an indication of the identification used in the study carried out, trade name, color index, composition and acronym.

Name	Commercial Pigment/Binders [62,63]	Colour Index Name	Manufacturer’s Composition	Acronym
CY	Chromium yellow pigment	77600	Lead chromate	PY34
MO	Molybdenum orange pigment	77629	Lead chromate, sulfate and molybdate	PR104
O	Ochre pigment	77492	Iron hydroxide	PY43
GE	Green earth pigment	77009	Iron (II) silicoaluminate, Mg and K	PG23
CG	Chromium green pigment	77288	Chromium oxide	PG15
NS	Natural sienna pigment	77491-2	Calcined natural iron oxide	PBr7
ZY	Zinc yellow pigment	77956	Zinc chromate	PY36
UB	Ultramarine blue pigment	77007	Sodium polysulfide-aluminosilicate	PB29

**Table 2:** List of raw materials used for binders along with an indication of the identification used in the study carried out, trade name, color index, composition and acronym.

Name	Commercial Pigment/Binders [62,63]	Colour Index Name	Manufacturer’s Composition	Acronym
P	Plaster of paris	77231	Hemihydrite	PW25
LW	Lime White	77220	Calcium hydroxide-Portlandite	PW18
NHL	Natural hydraulic lime	77230	Silica calcium aluminates and calcium hydroxide	PW28
WG	Water glass	77007	Sodium metasilicate	PB29

**Table 3:** Chemical composition by XRF analysis (wt %) of each raw material. Data normalized to 100% (LOI-free), loss on ignition.

SAMPLE (wt %)	SiO_2_	Al_2_O_3_	Fe_2_O_3_	MnO	MgO	CaO	Na_2_O	K_2_O	TiO_2_	P_2_O_5_	SO_3_	Cl	LOI
Plaster	0.508	0.402	0.212	0.108	0.602	32.205		0.047	0.196	0.021	45.450		20.106
Aerial lime	0.202		0.076		0.516	76.292	0.128	0.068		0.630	0.196	0.412	21.430
Hidraulic lime	12.908	4.052	1.902	0.028	0.944	58.653	0.098	0.925	0.247	0.052	0.093		19.918
Sodium silicate	48.09	0.18	0.03			0.03	19.57	0.08	0.01	0.01	0.05		31.78

**Table 4:** Specification and dosage of each gypsum-based paste mix (by volume %).

Name	Plaster	Aerial Lime	Hidraulic Lime	Sodium Silicate Solution (25–75%)	Pigment	Water Added
	%	%	%	%	%	parts
PPS	80	0	0		20	0.5
PALS	65	15	0		20	0.5
PHLS	65	0	15		20	0.5
PWGS	65			15	20	

**Table 5:** Results of the water vapor permeability test and standard deviation for the different white pastes tested.

WVP (kg/(m·Pa·s))·10^−12^								
	28 Days				120 Days			
PIGMENT	PPS	PALS	PWGS	PHLS	PPS	PALS	PWGS	PHLS
Binder	33.4 ± 1.50	27.2 ± 1.70	25.30 ± 0.40	22.8 ± 0.45	28.95 ± 1.35	25.89 ± 0.95	22.91 ± 0.83	20.96 ± 1.33
CY	33.1 ± 0.80	26.9 ± 1.10	24.90 ± 1.25	20.9 ± 1.17	31.4 ± 0.90	25.5 ± 0.95	23.6 ± 0.83	19.8 ± 1.33
MO	32.9 ± 1.10	26.5 ± 1.30	24.50 ± 1.10	20.5 ± 1.07	31.2 ± 1.30	25.1 ± 1.18	23.2 ± 1.13	19.4 ± 1.25
O	33.8 ± 1.70	26.9 ± 1.50	24.90 ± 1.33	21.1 ± 1.25	30.9 ± 0.80	25.5 ± 0.95	23.6 ± 1.06	20.5 ± 1.10
GE	32.3 ± 1.3	26.1 ± 1.20	24.10 ± 1.11	20.0 ± 1.50	30.5 ± 1.80	24.8 ± 0.91	22.9 ± 1.12	19.0 ± 1.30
CG	33.2 ± 0.70	27.4 ± 1.33	25.43 ± 1.20	21.5 ± 0.94	31.5 ± 1.20	26.3 ± 1.46	24.1 ± 1.38	20.4 ± 0.98
NS	32.3 ± 1.50	27.2 ± 0.95	25.72 ± 1.80	20.5 ± 1.37	32.1 ± 1.50	25.8 ± 1.13	23.9 ± 0.95	19.48 ± 1.80
ZY	33.3 ± 1.12	27.1 ± 1.17	25.47 ± 1.40	21.4 ± 1.22	31.6 ± 1.10	25.7 ± 1.20	23.8 ± 1.05	20.3 ± 1.10
UB	32.9 ± 1.13	26.5 ± 1.08	24.39 ± 1.18	20.5 ± 1.30	31.2 ± 1.10	25.1 ± 1.32	23.2 ± 0.88	19.8 ± 1.18

**Table 6:** Results of the mechanical tests (120 days) and standard deviation for the different pastes tested.

Samples	Compressive Strength (MPa)		Flexural Strength (MPa)	
		σ		σ
PP	5.40	0.02	2.25	0.03
PAL	5.62	0.04	2.93	0.06
PHL	9.79	0.03	3.90	0.05
PWG	8.65	0.01	3.51	0.04

**Table 7:** Results for mechanical tests and standard deviation for the different pastes containing added pigments tested after 120 days.

Samples	Compressive Strength (MPa)		Flexural Strength (MPa)	
PPS		σ		σ
PPS + CY	5.40	0.03	2.24	0.02
PPS + MO	5.42	0.05	2.25	0.04
PPS + O	5.43	0.04	2.24	0.03
PPS + GE	5.52	0.02	2.32	0.03
PPS + CG	5.42	0.03	2.26	0.02
PPS + NS	5.45	0.04	2.21	0.05
PPS + ZY	5.30	0.05	2.22	0.04
PPS + UB	5.36	0.03	2.23	0.03
PALS		σ		σ
PALS + CY	5.61	0.02	2.91	0.04
PALS + MO	5.64	0.05	2.92	0.05
PALS + O	5.63	0.04	2.93	0.06
PALS + GE	5.74	0.05	2.94	0.03
PALS + CG	5.56	0.03	2.91	0.06
PALS + NS	5.60	0.02	2.93	0.05
PALS + ZY	5.58	0.05	2.94	0.04
PALS + UB	5.62	0.03	2.92	0.02
PWGS		σ		σ
PWGS + CY	8.66	0.03	3.50	0.02
PWGS + MO	8.68	0.01	3.51	0.01
PWGS + O	8.67	0.02	3.50	0.06
PWGS + GE	8.72	0.01	3.52	0.04
PWGS + CG	8.68	0.04	3.52	0.05
PWGS + NS	8.71	0.05	3.48	0.03
PWGS + ZY	8.65	0.06	3.48	0.06
PWGS + UB	8.62	0.01	3.49	0.02
PHLS		σ		σ
PHLS + CY	9.80	0.02	3.92	0.05
PHLS + MO	9.76	0.04	3.91	0.06
PHLS + O	9.73	0.01	3.93	0.04
PHLS + GE	9.91	0.02	3.92	0.03
PHLS + CG	9.80	0.05	3.94	0.03
PHLS + NS	9.82	0.03	3.91	0.05
PHLS + ZY	9.84	0.03	3.92	0.02
PHLS + UB	9.85	0.03	3.90	0.07

**Table 8:** CIELab* 1976 values for the pigments and pastes used in this study and total color variations (ΔE), chromaticity variations (ΔC) and luminosity variations (ΔL) of the pigments used in the preparation of the pastes after 28 and 120 days.

	28D			120D			28D	120D	28D	120D	28D	120D
	a*	b*	L*	a*	b*	L*	ΔE	ΔE	ΔC	ΔC	ΔL	ΔL
PIGMENT CY	−17.03	92.10	98.40	−17.03	92.10	98.40	0.00	0.00	0.00	0.00	0.00	0.00
PPS + CY (80–20%)	−5.00	62.00	93.20	−3.99	31.09	95.02	−23.80	−23.84	−31.46	−62.32	−5.20	−3.38
PALS + CY (65–15–20%)	−5.00	63.12	92.80	−3.00	32.00	94.00	−23.51	−23.58	−30.34	−61.52	−5.60	−4.40
PHLS + CY (65–15–20%)	−6.00	62.00	91.03	−5.00	31.00	91.00	−25.55	−25.60	−31.37	−62.26	−7.37	−7.40
PWGS + CY (65–15–20%)	−9.00	61.00	92.00	−9.00	29.07	93.91	−25.10	−25.10	−32.00	−63.23	−6.40	−4.49
X	−6.25	62.03	92.26	−5.25	30.79	93.48	−24.49	−24.53	−31.29	−62.33	−6.14	−4.92
σ	1.89	0.87	0.96	2.63	1.23	1.73	0.99	0.97	0.69	0.70	0.96	1.73
PIGMENT MO	74.20	65.13	55.40	74.20	65.13	55.40	0.00	0.00	0.00	0.00	0.00	0.00
PPS + MO (80–20%)	33.40	18.41	67.09	16.14	6.09	81.88	−36.04	−41.79	−60.59	−81.48	11.69	26.48
PALS + MO (65–15–20%)	33.00	20.01	67.00	16.15	7.00	81.00	−35.89	−41.45	−60.14	−81.13	11.60	25.60
PHLS + MO (65–15–20%)	32.99	19.05	65.00	15.08	6.03	78.14	−37.87	−43.82	−60.63	−82.49	9.60	22.74
PWGS + MO (65–15–20%)	30.00	17.02	66.00	11.00	4.00	81.00	−38.74	−44.17	−64.24	−87.03	10.60	25.60
X	32.35	18.62	66.27	14.59	5.78	80.51	−37.14	−42.81	−61.40	−83.03	10.87	25.11
σ	1.58	1.25	0.98	2.45	1.27	1.63	1.40	1.39	1.91	2.72	0.98	1.63
PIGMENT NS	11.40	37.20	55.47	11.40	37.20	55.47	0.00	0.00	0.00	0.00	0.00	0.00
PPS + NS (80–20%)	5.00	16.99	71.80	2.79	5.88	85.00	6.20	6.08	−21.20	−32.40	16.33	29.53
PALS + NS (65–15–20%)	4.99	18.00	71.00	3.00	6.99	84.50	5.66	5.55	−20.23	−31.30	15.53	29.03
PHLS + NS (65–15–20%)	4.00	17.07	69.99	2.40	7.01	82.10	4.40	4.33	−21.38	−31.50	14.52	26.63
PWGS + NS (65–15–20%	1.99	16.04	71.09	−2.00	4.03	84.10	5.15	5.15	−22.74	−34.41	15.62	28.63
X	4.00	17.03	70.97	1.55	5.98	83.93	5.35	5.28	−21.39	−32.40	15.50	28.46
σ	1.42	0.80	0.74	2.38	1.40	1.27	0.77	0.74	1.04	1.42	0.74	1.27
PIGMENT GE	−6.00	10.00	59.15	−6.00	10.00	59.15	0.00	0.00	0.00	0.00	0.00	0.00
PPS + GE (80–20%)	−3.00	3.00	78.00	−1.01	0.30	87.88	17.83	17.78	−7.42	−10.61	18.85	28.73
PALS + GE (65–15–20%)	−3.00	4.00	77.00	−1.00	1.00	87.62	16.87	16.82	−6.66	−10.25	17.85	28.47
PHLS + GE (65–15–20%)	−2.99	3.02	76.13	−2.00	0.99	84.40	15.96	15.93	−7.41	−9.43	16.98	25.25
PWGS + GE (65–15–20%	−6.09	1.99	76.99	−6.07	−2.00	86.85	16.97	16.97	−5.26	−5.27	17.84	27.70
X	−3.77	3.00	77.03	−2.52	0.07	86.69	16.91	16.87	−6.69	−8.89	17.88	27.54
σ	1.55	0.82	0.76	2.41	1.42	1.59	0.76	0.76	1.02	2.46	0.76	1.59
PIGMENT CG	−25.30	19.02	47.04	−25.30	19.02	47.04	0.00	0.00	0.00	0.00	0.00	0.00
PPS + CG (80–20%)	^−12^.00	9.00	68.00	−5.00	2.00	83.00	12.94	12.08	−16.65	−26.27	20.96	35.96
PALS + CG (65–15–20%)	−11.99	8.99	67.04	−4.99	3.01	82.89	12.00	11.13	−16.67	−25.82	20.00	35.85
PHLS + CG (65–15–20%)	^−12^.00	9.00	66.30	−6.02	2.95	79.09	11.28	10.48	−16.65	−24.95	19.26	32.05
PWGS + CG (65–15–20%	-14.00	6.89	67.00	−10.01	0.09	82.07	12.10	11.40	−16.05	−21.64	19.96	35.03
X	^−12^.50	8.47	67.09	−6.51	2.01	81.76	12.08	11.27	−16.50	−24.67	20.05	34.72
σ	1.00	1.05	0.70	2.39	1.36	1.83	0.68	0.66	0.30	2.09	0.70	1.83
PIGMENT O	7.40	45.30	87.20	7.40	45.30	87.20	0.00	0.00	0.00	0.00	0.00	0.00
PPS + O (80–20%)	4.01	20.99	91.20	1.97	8.98	95.10	−4.87	−4.94	−24.53	−36.71	4.00	7.90
PALS + O (65–15–20%)	4.02	21.00	91.49	2.00	10.00	93.80	−4.59	−4.65	−24.52	−35.70	4.29	6.60
PHLS + O (65–15–20%)	4.00	20.98	89.04	2.00	9.00	91.25	−6.98	−7.04	−24.54	−36.68	1.84	4.05
PWGS + O (65–15–20%)	0.99	19.00	91.00	−2.94	5.99	93.01	−5.58	−5.53	−26.87	−39.23	3.80	5.81
X	3.26	20.49	90.68	0.76	8.49	93.29	−5.50	−5.54	−25.12	−37.08	3.48	6.09
σ	1.51	1.00	1.11	2.47	1.73	1.61	1.07	1.07	1.17	1.51	1.11	1.61
PIGMENT ZY	−10.99	78.08	88.10	−10.99	78.08	88.10	0.00	0.00	0.00	0.00	0.00	0.00
PPS + ZY (80–20%)	−8.00	48.00	91.00	−5.00	22.00	94.00	−15.04	−15.23	−30.19	−56.29	2.90	5.90
PALS + ZY (65–15–20%)	−8.00	47.99	90.10	−5.00	24.02	93.20	−15.84	−16.03	−30.20	−54.31	2.00	5.10
PHLS + ZY (65–15–20%)	−9.00	48.03	89.00	−6.00	23.20	91.40	−16.70	−16.92	−29.98	−54.89	0.90	3.30
PWGS + ZY (65–15–20%	−11.79	47.10	91.16	−9.99	21.04	93.00	−14.95	−15.14	−30.30	−55.56	3.06	4.90
X	−9.20	47.78	90.32	−6.50	22.57	92.90	−15.63	−15.83	−30.17	−55.26	2.22	4.80
σ	1.79	0.45	0.99	2.38	1.31	1.09	0.82	0.83	0.13	0.85	0.99	1.09
PIGMENT UB	36.60	−65.67	24.98	36.60	−65.67	24.98	0.00	0.00	0.00	0.00	0.00	0.00
PPS + UB (80–20%)	16.00	−37.99	59.00	8.00	−20.00	78.00	−7.25	−8.59	−33.96	−53.64	34.02	53.02
PALS + UB (65–15–20%)	15.99	−37.00	58.04	7.00	−19.04	77.08	−8.56	−10.04	−34.87	−54.89	33.06	52.10
PHLS + UB (65–15–20%)	16.01	−38.98	57.51	6.82	−20.04	75.90	−7.93	−9.41	−33.04	−54.01	32.53	50.92
PWGS + UB (65–15–20%	14.02	−39.40	58.70	3.00	−22.10	78.03	−7.15	−8.46	−33.36	−52.88	33.72	53.05
X	15.51	−38.34	58.31	6.21	−20.30	77.25	−7.72	−9.13	−33.81	−53.86	33.33	52.27
σ	0.99	1.07	0.67	2.20	1.29	1.00	0.66	0.74	0.81	0.84	0.67	1.00

## Data Availability

Not applicable.
